# Biophysical screening methods for extracellular domain peptide receptors, application to natriuretic peptide receptor C ligands

**DOI:** 10.1111/cbdd.13395

**Published:** 2018-10-10

**Authors:** Daniel Conole, Samuel H. Myers, Filipa Mota, Adrian J. Hobbs, David L. Selwood

**Affiliations:** ^1^ Wolfson Institute for Biomedical Research University College London London UK; ^2^ William Harvey Research Institute Heart Centre, Barts & The London School of Medicine Queen Mary University of London London UK

**Keywords:** biophysical, CNP, fluorescence polarization, NPR‐C, surface plasmon resonance, thermal shift

## Abstract

Endothelium‐derived C‐type natriuretic peptide possesses cytoprotective and anti‐atherogenic functions that regulate vascular homeostasis. The vasoprotective effects of C‐type natriuretic peptide are somewhat mediated by the natriuretic peptide receptor C, suggesting that this receptor represents a novel therapeutic target for the treatment of cardiovascular diseases. In order to facilitate our drug discovery efforts, we have optimized an array of biophysical methods including surface plasmon resonance, fluorescence polarization and thermal shift assays to aid in the design, assessment and characterization of small molecule agonist interactions with natriuretic peptide receptors. Assay conditions are investigated to explore the feasibility and dynamic range of each method, and peptide‐based agonists and antagonists are used as controls to validate these conditions. Once established, each technique was compared and contrasted with respect to their drug discovery utility. We foresee that such techniques will facilitate the discovery and development of potential therapeutic agents for NPR‐C and other large extracellular domain membrane receptors.

## Graphical abstract

C‐type natriuretic peptide and its cognate receptor, natriuretic peptide receptor C, represent a novel therapeutic target for the treatment of cardiovascular diseases. To facilitate our drug discovery efforts, we have optimized an array of biophysical methods including surface plasmon resonance, fluorescence polarization and thermal shift assays to aid in the design, assessment and characterization of small molecule agonist interactions with natriuretic peptide receptors.

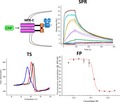

**Abbreviations**
AC: adenylate cyclase
CNP: C‐type natriuretic peptide
ECD: extracellular domain
FL: full length
GIRK: G‐protein‐coupled inwardly rectifying potassium channels
Giα,β,Ƴ: G (guanine nucleotide‐binding) proteins and their alpha, beta and gamma subunits
NPR‐C: natriuretic peptide receptor C
PLCβ: phospholipase Cß
SDS‐PAGE: sodium dodecyl sulphate polyacrylamide gel electrophoresis
SM: smooth muscle

## 1 INTRODUCTION

Endothelium‐derived C‐type natriuretic peptide (CNP) possesses cytoprotective and anti‐atherogenic functions that regulate vascular homeostasis.^1^, ^2^, ^3^ The vasoprotective effects of CNP are mediated, at least in part, by natriuretic peptide receptor type‐C (NPR‐C), which is widely thought to function as a systemic clearance receptor, removing natriuretic peptides from the circulation through internalization and degradation.^4^, ^5^

**Figure 1:**
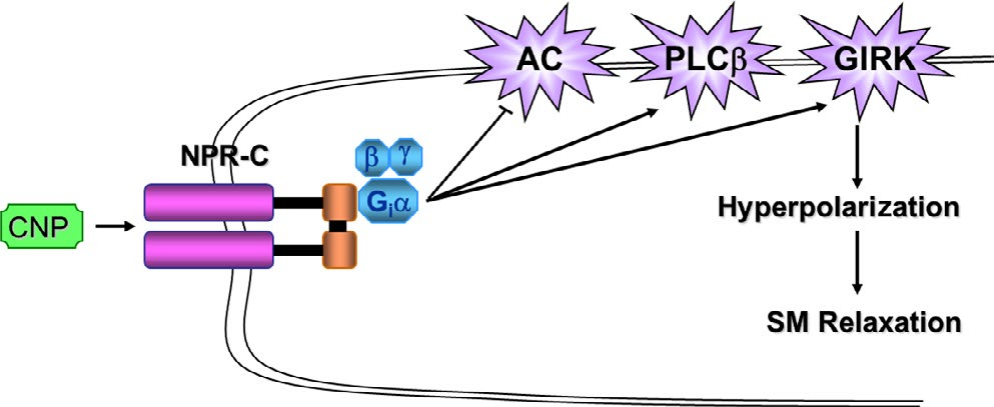
Schematic representation of the key cell signalling activities of natriuretic peptide receptor C (NPR‐C) activation.^7^ C‐type natriuretic peptide binds the NPR‐C receptor and causes a conformational change that allows Giα to bind and then inhibit adenylate cyclase enzyme. Similarly, NPR‐C may modulate phospholipase Cβ via the βγ subunit.^8^ Gi protein activation also produces vascular smooth muscle relaxation through interaction with G‐protein‐coupled inwardly rectifying potassium channels [Colour figure can be viewed at http://www.wileyonlinelibrary.com/]

C‐type natriuretic peptide produces vascular smooth muscle (SM) relaxation via NPR‐C agonism, stimulating Gi protein and G‐protein‐coupled inwardly rectifying potassium channels (GIRKs), resulting in hyperpolarization (Figure 1).^6^ Furthermore, administration of small molecule NPR‐C agonists exerts a dose‐dependent hypotension in vivo.^1^

Despite sophisticated in vivo characterization of the modulatory effects of CNP and a handful of small molecule agonists on NPR‐C, no practical and robust screening cascade exists to identify and characterize more potent, bioavailable small molecule NPR‐C agonists in vitro. In this work, we utilize biophysical assays such as surface plasmon resonance,^9^ fluorescence polarization^10^ and thermal shift^11^ to establish and optimize a reliable in vitro screening platform to allow NPR‐C receptor ligands to be identified, selected and ranked for more advanced functional in vivo assays.

## 2 METHODS AND MATERIALS

### 2.1 Materials

The selective NPR‐C antagonist M372049^12^ (WuXi AppTec, China) and CNP (Sigma‐Aldrich, UK) were obtained commercially. Dual NPR‐A and NPR‐C ligand PL‐3994 was a gift from the Hobbs Lab at QMUL.^13^ Human full‐length (FL) NPR‐C (27–541)‐10His‐Flag (GenBank accession: NM_000908) and extracellular domain (ECD) NPR‐C (27–481)‐6His were expressed and isolated at Peak Proteins, UK. The fluorescent reporter molecule, 5‐carboxyfluorescein *N*‐terminus labelled GLSKG[CFGRSLDRIGSLSGLGC]NS (Flu‐P19), GLSKG(CFVNVSQDRIGSQSGLGC)NS (Flu‐P20) and GLSKG(CFGRSLDRIGSLSGSGC)TQDS (Flu‐P21) were obtained commercially (Peptide Protein Research Ltd, UK). The Protein Thermal Shift^™^ Dye Kit was obtained commercially (Thermo Fisher Scientific, UK).

### 2.2 Protein characterization and oligomer status

Conditions for full‐length NPR‐C sodium dodecyl sulphate polyacrylamide gel electrophoresis (SDS‐PAGE): Novex Wedgewell 10%–20% Tris‐Glycine 12 well (Invitrogen XP00102); markers: RunBlue prestained tricolour marker, Expedion NXA6050, 4 μl loaded (MW); either 4, 2 or 1 μg protein loaded under reducing (R) or non‐reducing (NR) conditions. Conditions for extracellular NPR‐C SDS‐PAGE: Novex Wedgewell 10%–20% Tris‐Glycine 12 well (Invitrogen XP00102); markers: RunBlue prestained tricolour marker, Expedion NXA6050, 5 μl loaded; either 3 or 5 μg protein loaded under reducing or non‐reducing conditions.

### 2.3 Surface plasmon resonance

All SPR analysis was performed on a BIAcore T200 system using Series S CM5 sensor chips. All sensorgrams were double‐referenced by subtracting the response on a reference flow cell and a blank sample. Ligands were evaluated against both the FL (27–541) and ECD (27–481) of NPR‐C. Human FL NPR‐C (27–541)‐10His‐Flag and ECD NPR‐C (27–481)‐6His were covalently attached to a CM5 chip via amine coupling^14^ with a surface density of 10,000 RU and 5,000 RU, respectively. Single‐cycle sequential injections of CNP (0.5–8 nM) were performed at a flow rate of 30 μl/min (240 s for each), followed by a dissociation time of 3,600 s. Binding of M372049 (0.23–500 nM) and PL‐3994 (8.3 pM–500 nM) was analysed by multi‐cycle sequential injections (90‐s association time for both M372049 and PL‐3994) followed by undisturbed dissociation (240 s for both M372049 and PL‐3994), during which curves returned to baseline. Peptide stocks were dissolved in dimethyl sulfoxide (DMSO), and the final sample solutions for kinetic affinity experiments contained 1% DMSO in 1× phosphate‐buffered saline P20 buffer (PBS‐P, Cat no 28995084, GE Healthcare Ltd.). DMSO solvent effects were corrected for with eight calibration solutions (0.5%–1.8% DMSO in PBS‐P). Equilibrium constants (*K*_D_) were calculated using either kinetic or affinity models, assuming simple 1:1 (Langmuir) binding. Data processing and analysis were performed using BIAevaluation and OriginPro software. The theoretical *R*_max_ (the maximal feasible signal between a ligand–analyte pair) for each compound/protein pair was calculated using Equation (1):^14^

(1) $$R_{\max}=R_{\mathrm{ligand}}\cdot \left(\frac{{\text{Mr}}_{\mathrm{analyte}}}{{\text{Mr}}_{\mathrm{ligand}}}\right)\cdot V_{\mathrm{ligand}},$$

where *R*_ligand_, amount of protein loaded in the SPR chip in response units; Mr_analyte_, molecular weight of the compound of interest; Mr_ligand_, molecular weight of the immobilized protein; *V*_ligand_, stoichiometry of the binding interaction between the ligand and the analyte.

The experimentally observed *R*_max_ was then calculated as a percentage of the theoretical *R*_max_ as a quality control measure. An experimental *R*_max_ <100% of the theoretical *R*_max_ was considered sensible and indicative of genuine binding.^14^

### 2.4 Fluorescence polarization

All experiments were performed using HEPES buffer, an 80 μl final volume and 3% DMSO. Protein titration experiments (0.12–500 nM) were performed at 15 nM probe concentration, selected with guidance from in‐house SPR analysis and the literature *K*_i_ of the untagged P19 peptide.^15^ To achieve a balance of protein consumption and fluorescence polarization dynamic read‐out range for competition experiments, the concentrations selected for FL and extracellular NPR‐C proteins were 150 and 400 nM. Keeping the appropriate reaction plate or tubes on ice, the optimized fluorescence polarization samples were prepared in the following order—ligand in HEPES buffer (CNP or M372049, 20 μl), NPR‐C protein (600 nM, FL, 20 μl, or 1,600 nM, ECD, 20 μl) in HEPES buffer and Flu‐P19 probe (30 nM, 40 μl) in HEPES buffer. Fluorescence polarization competition experiments against the Flu‐P19 probe were performed with varying concentrations (4.89 nM–2.5 μM) of each CNP and M372049 and their fluorescence polarization read‐out measured and normalized to experiment controls (buffer + probe, buffer + probe + protein) using a BMG Labtech Omega Pherastar^®^ plate reader (filter settings: 485 nm [excitation] and 520 nm [emission]). Background fluorescence polarization was blanked using a HEPES buffer‐only control. Raw data were processed using OriginPro curve fitting software to obtain the IC_50_s. A web‐based IC_50_‐to‐ *K*_i_ converter that computes *K*_i_ values from experimentally determined IC_50_ values was employed.^16^

### 2.5 Thermal shift

Thermal shift assays were performed using Thermo Fisher Scientific Protein Thermal Shift^™^ Dye Kit, and samples were prepared according to the accompanying protocol.^17^ A solution of Protein Thermal Shift^™^ Dye was always prepared fresh from the 1,000× stock. Optimization experiments were performed by titrating NPR‐C protein (0.1, 0.05 and 0.01 mg/ml) and Protein Thermal Shift^™^ Dye concentration (80, 8 and 1×) to identify conditions that achieved melt curves with the greatest dynamic fluorescence range and least standard error in *T*_m_ (melt temperature) values. Keeping the reaction plate on ice, the optimized protein melt reactions were prepared in the following order—Protein Thermal Shift^™^ Buffer (5.0 μl), water + FL NPR‐C (0.45 mg/ml stock, 4.44 μl) + buffer or ligand (CNP, M372049, PL‐3994, 1:1 or 10:1 stoichiometric ratios with the protein, 12.5 μl) and Diluted Protein Thermal Shift^™^ Dye (8× stock 2.5 μl). The total volume for each reaction was 20.0 μl. Each melt reaction was mixed at least 10 times, and the plate was sealed with MicroAmp^®^ Optical Adhesive Film. The plate was then spun it at 112 *g* for 1 min and placed on ice until measurement. Thermal melt reactions were performed and analysed using a 7500 Real‐Time PCR System^®^. A 5‐carboxy‐X‐rhodamine (ROX) reporter and a continuous temperature ramp of 25–99°C were used (2 min per step). Optical filters were set at 580 nm (excitation) and 623 nm (emission). The *T*_m_ for each sample was obtained by differentiating the original fluorescence melt curve using OriginPro mathematical and peak identification functions. The reported *T*_m_ values are the mean across four replicates.

## 3 RESULTS

### 3.1 Protein characterization and oligomer status

**Figure 2:**
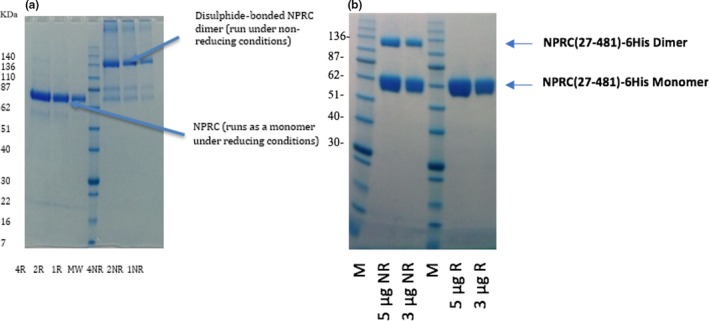
Reducing and non‐reducing sodium dodecyl sulphate polyacrylamide gel electrophoresis for full‐length natriuretic peptide receptor C (NPR‐C) (a) (27–541) and extracellular domain NPR‐C (b) (27–481) [Colour figure can be viewed at http://www.wileyonlinelibrary.com/]

Non‐reducing SDS‐PAGE analyses of the protein batches used in these experiments indicated that FL NPR‐C was primarily present as the active disulphide‐bonded dimer (~130 kDa), whereas the ECD mainly existed as the monomer (~55 kDa; Figure 2).

### 3.2 Surface plasmon resonance

**Figure 3:**
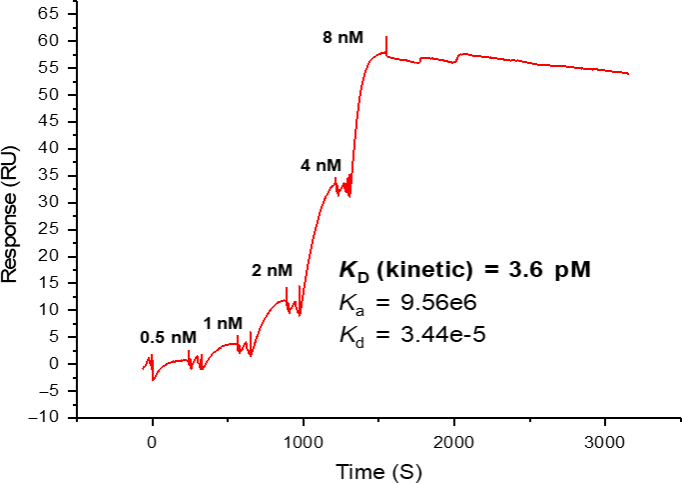
Single‐cycle kinetics (0.5–8 nM, 5 concentrations) experiment with C‐type natriuretic peptide against full‐length natriuretic peptide receptor C (27–541). *K*_a_ (1/Ms) is the association constant, and *K*_d_ (1/s) is the dissociation constant—the *K*_D_ is calculated from these parameters using the Langmuir 1:1 binding model (see Section 2 for further details) [Colour figure can be viewed at http://www.wileyonlinelibrary.com/]

**Figure 4:**
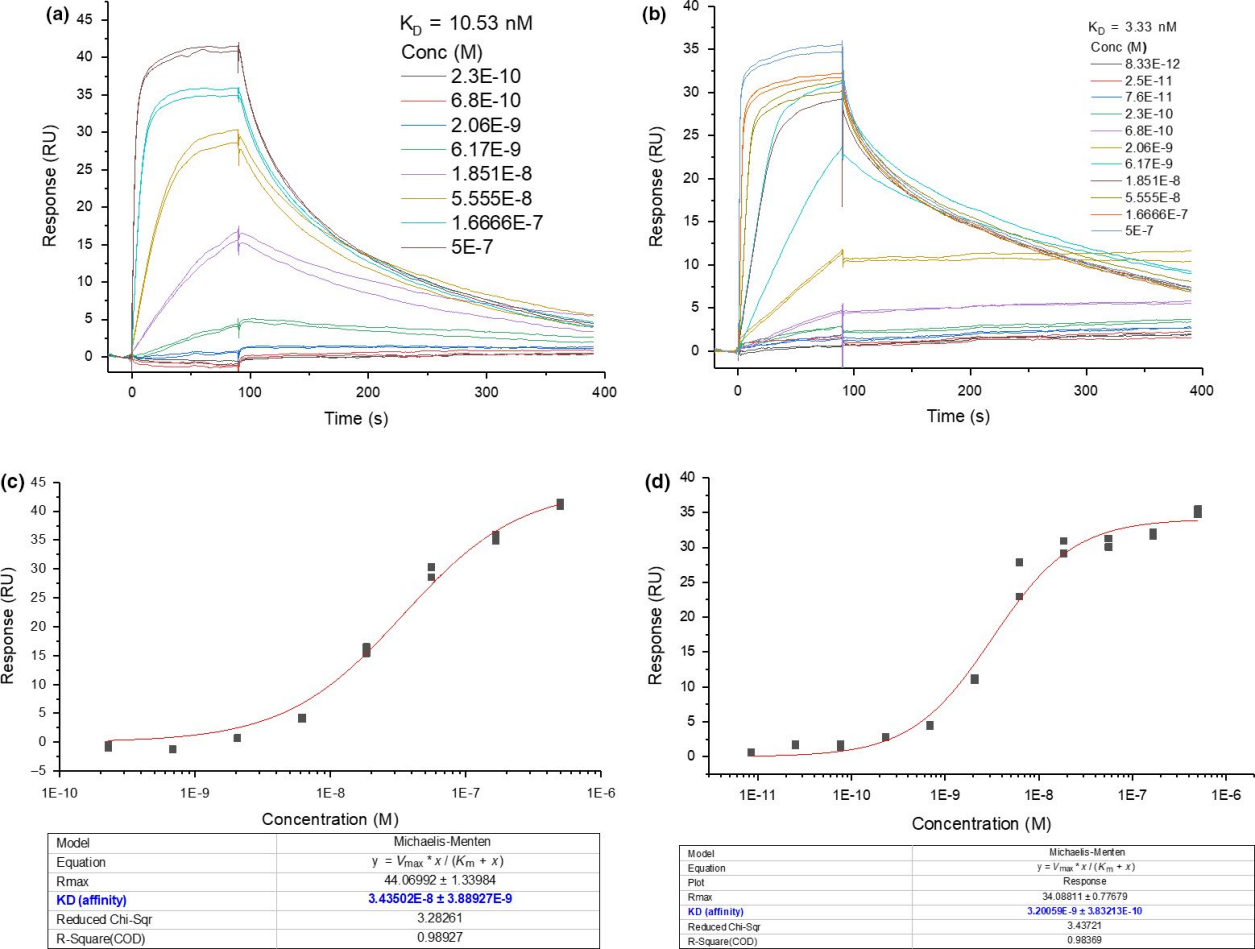
Representative raw sensorgrams for M372049 (a) and PL‐3994 (b). Natriuretic peptide receptor C (NPR‐C) peptide binders. *K*_D_ values (equilibrium constants) were calculated using the kinetic (Langmuir 1:1) model. Representative steady‐state affinity curves for M372049 (**C**) and PL‐3994 (d) NPR‐C peptide binders. *K*_D_ values (equilibrium constants) were derived from a Michaelis–Menten model, which best fits the data [Colour figure can be viewed at http://www.wileyonlinelibrary.com/]

To validate the integrity of the NPR‐C protein and commercial peptides in our hands, SPR analyses of CNP and peptidomimetic NPR‐C antagonist M372049 were conducted and compared to that in the literature.^1^ Another NPR‐C binding peptide PL‐3994 was also evaluated, and all three *K*_D_ values were in agreement with the literature binding data 13 (Figures 3 and 4). CNP was evaluated using a single‐cycle kinetic run, due to its picomolar binding (Figure 3) preventing equilibrium binding analysis. Affinity curves for M372049 and PL‐3994 multi‐cycle runs exhibited sigmoidal curve shape and sensical *R*_max_ values, suggesting genuine NPR‐C binding saturation (Figure 4, Table 1).^18^ To illustrate the reliability of the protocol in our hands, peptide controls were run at least twice and were shown to be reproducible. We were interested to evaluate whether there was any difference in ligand binding between the dimeric FL and monomeric ECD immobilized NPR‐C. Interestingly, very similar data were obtained (Table 1).

**Table 1:** Summary of reproducibility of control peptide SPR analysis

Compound	NPR‐C	*K*_D_ (nM, affinity)	*K*_D_ (nM, kinetic)	% theoretical *R*_max_
CNP	(27–541)‐10His (FL)	–	0.0036	31
	(27–481)‐6His (ECD)	–	0.0069	51
M372049	(27–541)‐10His (FL)	34.35	10.53	59
		20.30	6.71	43
		16.50	8.08	36
	(27–481)‐6His (ECD)	22.23	13.08	63
		25.20	14.10	54
		22.10	13.7	52
PL‐3994	(27–541)‐10His (FL)	12.23	3.53	20
		3.33	0.65	21
		4.28	0.94	22
	(27–481)‐6His (ECD)	3.84	1.03	21
		4.89	1.20	21

*Notes*

FL: full length; ECD: extracellular domain; His: histidine; *K*_D_: equilibrium constant as calculated by either steady‐state affinity or kinetic methods; % theoretical *R*_max_: experimentally measured percentage of the maximal feasible signal between a ligand–analyte pair.

### 3.3 Fluorescence polarization

**Figure 5:**
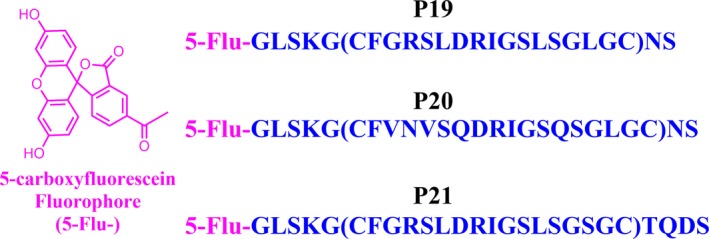
Peptide sequence and fluorophore structure of 5‐carboxyfluorescein *N*‐terminus labelled probes P19, P20 and P21 [Colour figure can be viewed at http://www.wileyonlinelibrary.com/]

**Figure 6:**
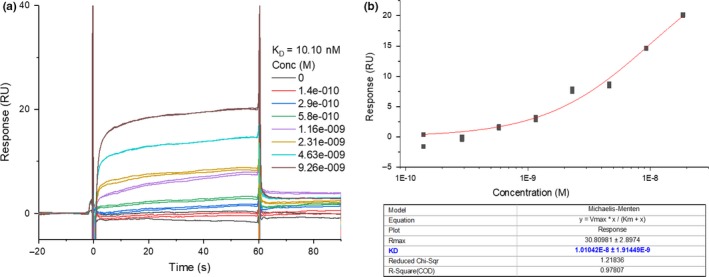
SPR raw sensorgram (a) and affinity curve (b) for Flu‐P19 binding to full length natriuretic peptide receptor C [Colour figure can be viewed at http://www.wileyonlinelibrary.com/]

**Figure 7:**
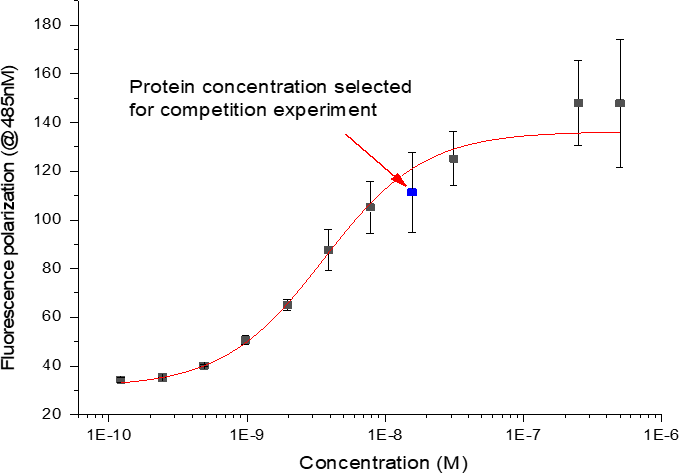
Titration of full‐length natriuretic peptide receptor C protein against a fixed concentration (15 nM) of Flu‐P19 probe. The protein concentration selected was 150 nM [Colour figure can be viewed at http://www.wileyonlinelibrary.com/]

Using phage display methodology, Deschênes et al.^15^ identified potent and selective peptide antagonists of NPR‐B. It was also discovered that a number of these peptides, termed P19, P20 and P21, displayed competitive inhibition of NPR‐C against radiolabelled ^125^I‐ANP in at least the nanomolar range.^15^ We postulated that one of these peptides may be a reasonable starting point to synthesize a fluorescent reporter molecule and commissioned the syntheses of their corresponding 5‐carboxyfluorescein N‐terminus tagged derivatives (Figure 5). Steady‐state affinity SPR analysis of the Flu‐P19 probe demonstrated a *K*_D_ ~ 10 nM against FL NPR‐C (Figure 6). Based on this information and FP guidelines,^10^ we conducted a protein titration experiment at 15 nM Flu‐P19 probe concentration. The results exhibited a sigmoidal relationship, and to achieve a balance of protein consumption and FP dynamic read‐out range, an NPR‐C protein concentration of 150 nM was selected for competitive inhibition experiments with our three positive controls (Figure 7). While similar experiments were performed with the P20‐ and P21‐tagged peptides, only minimal fluorescence polarization windows could be obtained (data not shown).

**Figure 8:**
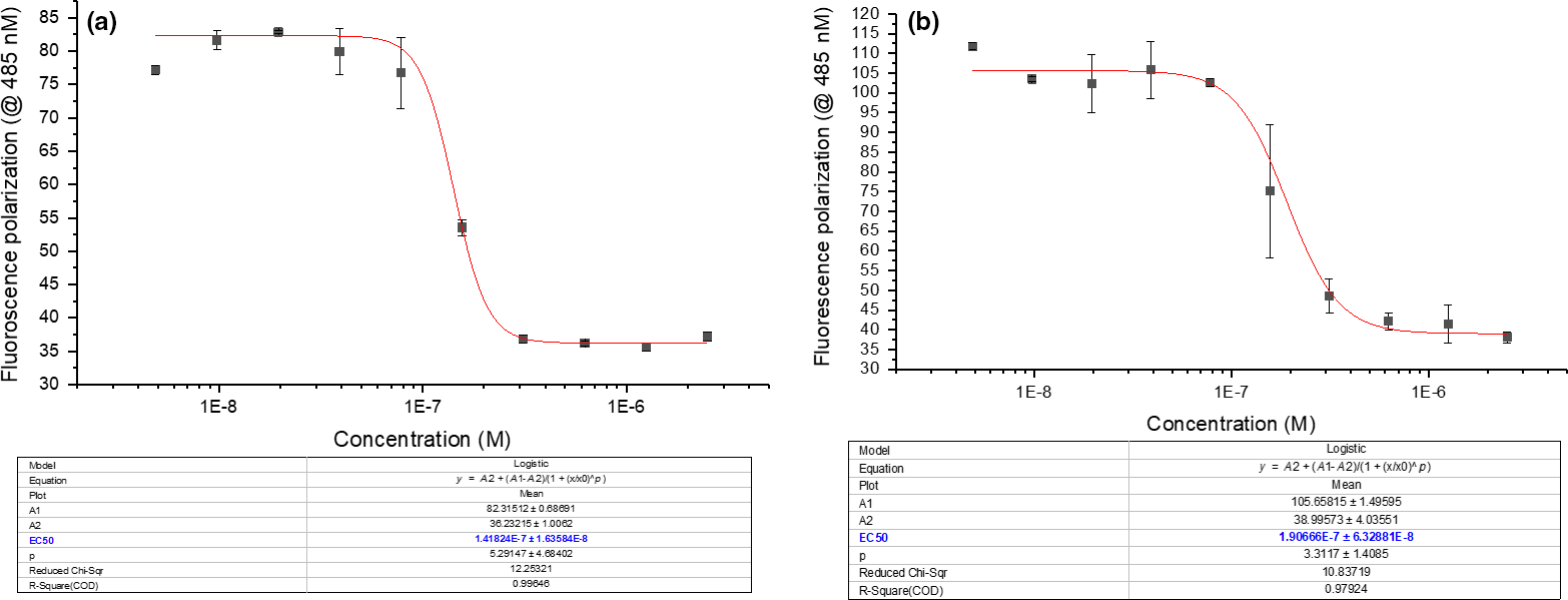
Representative fluorescence polarization competition experiments and calculated IC_50_ values between the fluorescently tagged Flu‐P19 peptide (15 nM) and natriuretic peptide receptor C peptide ligands C‐type natriuretic peptide (a) and M372049 (b) [Colour figure can be viewed at http://www.wileyonlinelibrary.com/]

Competition experiments with CNP and M372049 elicited IC_50_ values of 141.7 and 190.7 nM, respectively (Figure 8). Using optimized conditions, these experiments were also demonstrated to be reproducible across different days (Table 2). Evaluation of these ligands with the ECD of NPR‐C yielded similar results (Table 2). An important parameter to determine the robustness of a competition inhibition assay is the inhibition constant (*K*_i_). Using kinetic equations,^16^ *K*_i_'s (competitive) of 0.49 and 3.7 nM were calculated for CNP and M372049, respectively, which correlated (within 1–1.5 log concentration units) with that in the literature (Table 2).^13^, ^19^

**Table 2:** Summary of control peptide FP analyses

Compound	NPR‐C	IC_50_ (nM)	*K*_i_ (competitive; nM)
CNP	(27–541)‐10His (FL)	141.8	0.10 (Lit.^20^ 0.023)
		110.9	
		68.4	
	(27–481)‐6His (ECD)	54.9	
		60.3	
M372049	(27–541)‐10His (FL)	190.7	2.6 (Lit.^12^, ^19^ 21)
		236.0	
	(27–481)‐6His (ECD)	145.0	

*Notes*

*K*_i_ values were calculated using a web‐based IC_50_‐to‐*K*_i_ converter from experimentally determined IC_50_ values and compared to that in the literature.^16^

FL: full length; ECD: extracellular domain.

### 3.4 Thermal shift

**Figure 9:**
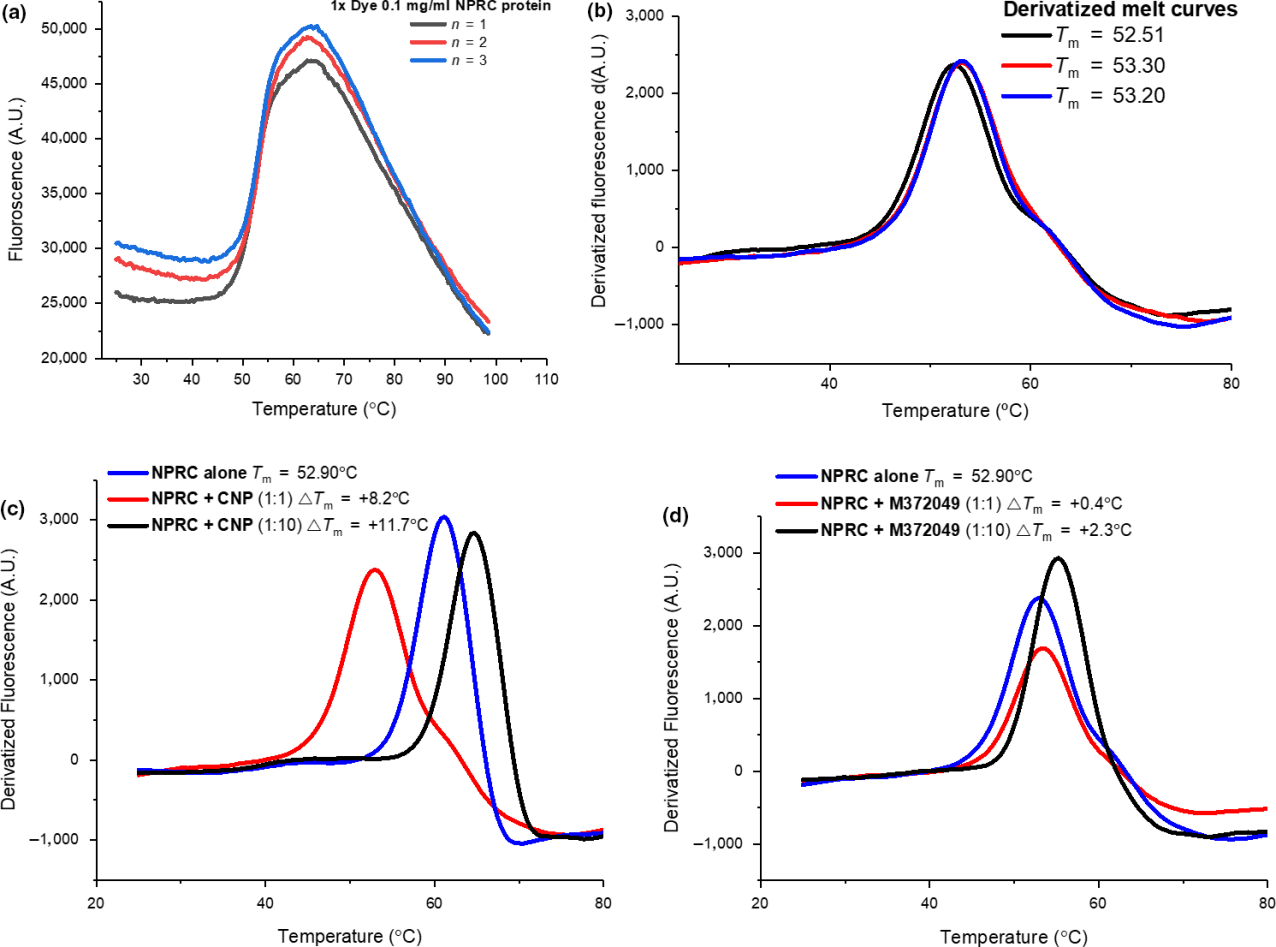
Raw (a) and differentiated (b) full length (FL) natriuretic peptide receptor C (NPR‐C) protein melt curves. Derivatized (differentiated) FL NPR‐C protein melt curves for increasing concentrations of agonist C‐type natriuretic peptide (c) and antagonist M372049 (b) [Colour figure can be viewed at http://www.wileyonlinelibrary.com/]

To our current knowledge, there is no literature for the determination of NPR‐C binding by thermal shift. This biophysical technique could serve as a useful orthogonal assay for confirmation of binding particularly for small molecules that bind non‐competitively with CNP‐like peptides. Using a commercial thermal shift assay kit, an assay condition optimization experiment was conducted and demonstrated that concentrations of 0.1 mg/ml of NPR‐C protein and 1× fluorescent dye produced consistent melt curves and accurate calculation of the NPR‐C *T*_m_ value (Figure 9a,b).

Using concentrations of CNP and M372049 as recommended by the literature^11^ and based on our optimized protein concentration, the effect of ligand treatment on NPR‐C *T*_m_ was investigated. Each of the peptide controls shifted the *T*_m_ by significant amounts, and the peptide agonist CNP gave a pronounced shift ΔTm ~11°C, whereas the antagonist M372049 gave a shift of Δ*T*_m_ ~2.5°C (Figure 9c,d).

## 4 DISCUSSION

Isolated G‐protein‐coupled receptors (GPCRs) are notoriously difficult to interrogate, as their active conformation is usually dependent on being supported in a membrane context.^21^ Characterization of NPR‐C binding in three distinct biophysical assays demonstrates that it is indeed possible to evaluate GPCRs without the need for complex membrane structure surrogates, such as nanodiscs.^22^, ^23^, ^24^ It should be noted however that NPR‐C is an atypical GPCR in that it lacks a seven transmembrane domain, and ~90% of this protein is extracellular.^25^, ^26^, ^27^, ^28^ These methods may not only be applicable for GPCRs but for other dissimilar receptors that possess a large and well‐defined ECD.^29^ We have shown this to be the case in the application of biophysical screening techniques for discovery of neuropilin‐1 ligands, an immunoglobulin domain protein receptor for the vascular endothelial growth factor (VEGF).^30^, ^31^, ^32^

The higher prevalence of the FL NPR‐C dimer compared with the ECD could be attributed to the proximity of the disulphide bond to the membrane.^33^ For the ECD, this suggested that only ~25% of the molecules are held together by disulphide bonds, and the non covalent dimers fell apart into monomers on the reducing SDS‐PAGE. This could have been anticipated, as the structural information on the FL NPR‐C shows extensive non covalent interactions contributing to the formation of the dimer.^34^ Interestingly, all three peptide ligands demonstrated similar binding kinetics for the FL and ECD NPR‐C, suggesting that the dimer formation is not necessarily required for active site binding.

The surface plasmon resonance analysis of the three peptides displayed highly sensitive binding detection, with distinct responses above the baseline observed at concentrations as low as 1 nM (Figures 3 and 4). It was interesting to observe that both dimeric FL and monomeric ECD NPR‐C possess similar properties with respect to the binding of ligands tested in this study. This suggests that surface plasmon resonance could be a useful tool for interrogating mutant NPR‐C proteins to gain further understanding of key binding residues. While surface plasmon resonance provides high information content, its sequential run operation somewhat limits its utility as a high‐throughput screen, at least when measuring concentration–response curves. In contrast, once a 384‐well fluorescence polarization plate has been prepared, spectroscopic read‐out of the entire plate takes place in a matter of minutes, suggesting this technique is more amenable to high‐throughput compound screening. The fluorescence polarization technique is limited, however, to interrogate molecules that compete with the reporter molecule for the orthosteric binding site, and so this particular assay set‐up would not be able to identify non‐competitive, allosteric modulators. In order to remedy this, an FP probe would need to be designed to bind at the identified allosteric site of interest, and while this would be challenging for this particular project, it has been achieved with other examples.^35^, ^36^ The thermal shift technique is equally amenable to high throughput, as plate reading takes only just over an hour; however, this technique is rather protein‐intensive and thus more expensive. The full contrast and comparison of the three biophysical methods compared with traditional radiolabelled ligand binding studies are outlined in Table 3. The choice of the most appropriate screening cascade will depend on the structural class and binding mode of the small molecules of interest. We propose that a combination of either surface plasmon resonance or fluorescence polarization as a primary screen, followed by a thermal shift assay to confirm binding, would function well as a mechanism to identify NPR‐C ligands that could go forward into more advanced functional in vivo assays to characterize agonists or antagonists.

**Table 3:** A contrast and comparison of three modern biophysical binding assays with respect to their utility in drug discovery compared with traditional radiolabelled ligand binding assays

Biophysical method	Advantages	Disadvantages
Surface plasmon resonance	High information content—binding constants (affinity and kinetics) can be determined Can evaluate multiple proteins binding simultaneously Minimal protein required Highly sensitive	Standard BIAcore machine is expensive, though cheaper alternatives may exist Medium throughput Cost of some consumables is high (microchips) Susceptible to false positives
Fluorescence polarization	High throughput Can determine EC_50_/*K*_i_ values for compound ranking	Detection of inhibitors that are allosteric require bespoke FP probe design and synthesis
Thermal Shift	High throughput Cheap Less susceptible to false positives	High protein concentration required False negatives
Traditional radiolabelled ligand binding assay	Highly sensitive	Uses hazardous materials Labour intensive (multiple washing steps) Expensive

## 5 CONCLUSIONS

We have established three biophysical methods and a framework to discover and characterize novel ligands of NPR‐C, an important protein for vascular homeostasis. Some of these methods are high throughput and can be adapted for library screening, whereas others could serve as an orthogonal biophysical technique. We foresee that such techniques will facilitate the discovery and development of potential therapeutic agents for cardiovascular diseases, and other extracellular membrane targets.

## CONFLICT OF INTEREST

DC, SM and FM declare that they have no conflict of interest. AH and DS are named inventors on a patent (EP3145914A1) covering the composition of novel NPR‐C agonists. Through the university revenue‐sharing scheme, inventors may, should the invention ever be commercialized, and in the fullness of time, receive some financial benefit.

## References

[1] A. J. Moyes , R. S. Khambata , I. Villar , K. J. Bubb , R. S. Baliga , N. G. Lumsden , F. Xiao , P. J. Gane , A. S. Rebstock , R. J. Worthington , M. I. Simone , F. Mota , F. Rivilla , S. Vallejo , C. Peiró , C. F. Sánchez Ferrer , S. Djordjevic , M. J. Caulfield , R. J. MacAllister , D. L. Selwood , A. Ahluwalia , A. J. Hobbs , J. Clin. Invest. 2014, 124, 4039. 25105365 10.1172/JCI74281 PMC4151218

[2] K. Nakao , K. Kuwahara , T. Nishikimi , Y. Nakagawa , H. Kinoshita , T. Minami , Y. Kuwabara , C. Yamada , Y. Yamada , T. Tokudome , C. Nagai‐Okatani , N. Minamino , Y. M. Nakao , S. Yasuno , K. Ueshima , M. Sone , T. Kimura , K. Kangawa , K. Nakao , Hypertension 2017, 69, 286. 28049696 10.1161/HYPERTENSIONAHA.116.08219

[3] B. Pressure , P. Eder‐negrin , R. H. Adams , H. A. Baba , Circulation 2018, 138, 494 10.1161/CIRCULATIONAHA.117.033383 29626067

[4] T. Maack , M. Suzuki , F. A. Almeida , D. Nussenzveig , R. M. Scarborough , G. A. McEnroe , J. A. Lewicki , Science (80‐.) 1987, 238, 675. 10.1126/science.2823385 2823385

[5] T. Maack , Kidney Int. 1996, 49, 1732. 8743487 10.1038/ki.1996.257

[6] S. D. Chauhan , H. Nilsson , A. Ahluwalia , A. J. Hobbs , Proc. Natl Acad. Sci. USA 2003, 100, 1426. 12552127 10.1073/pnas.0336365100 PMC298789

[7] R. A. Rose , W. R. Giles , J. Physiol. 2008, 586, 353. 18006579 10.1113/jphysiol.2007.144253 PMC2375602

[8] M. B. Anand‐Srivastava , Peptides 2005, 26, 1044. 15911072 10.1016/j.peptides.2004.09.023

[9] S. G. Patching , Biochim. Biophys. Acta ‐ Biomembr. 2014, 1838, 43. 10.1016/j.bbamem.2013.04.028 23665295

[10] N. J. Moerke , Curr. Protoc. Chem. Biol. 2009, 1, 1. 23839960 10.1002/9780470559277.ch090102

[11] M. Vivoli , H. R. Novak , J. A. Littlechild , N. J. Harmer , J. Vis. Exp. 2014, 91, 1. 10.3791/51809 PMC4692391 25285605

[12] I. C. Villar , C. M. Panayiotou , A. Sheraz , M. Madhani , R. S. Scotland , M. Nobles , B. Kemp‐Harper , A. Ahluwalia , A. J. Hobbs , Cardiovasc. Res. 2007, 74, 515. 17391657 10.1016/j.cardiores.2007.02.032 PMC3503309

[13] J. D. Edelson , M. Makhlina , K. R. Silvester , S. S. Vengurlekar , X. Chen , J. Zhang , C. J. Koziol‐White , P. R. Cooper , T. J. Hallam , D. W. Hay , R. A. Panettieri Jr , Pulm. Pharmacol. Ther. 2013, 26, 229. 23154072 10.1016/j.pupt.2012.11.001 PMC4070431

[14] A. B. Biacore , E. July , A. B. Biacore , BIAevaluation Software Handbook (3rd ed), Biacore AB, Uppsala, 1997.

[15] J. Deschênes , C. Duperé , N. McNicoll , N. L'Heureux , F. Auger , A. Fournier , A. De Léan , Peptides 2005, 26, 517. 15652659 10.1016/j.peptides.2004.10.017

[16] R. Z. Cer , U. Mudunuri , R. Stephens , F. J. Lebeda , Nucleic Acids Res. 2009, 37, 441. 19395593 10.1093/nar/gkp253 PMC2703898

[17] Applied Biosystems , Protein Thermal Shift ™ Studies, Life Technologies, Carlsbad, CA 2011.

[18] A. M. Giannetti , B. D. Koch , M. F. Browner , J. Med. Chem. 2008, 51, 574. 18181566 10.1021/jm700952v

[19] C. A. Veale , V. C. Alford , D. Aharony , D. L. Banville , R. A. Bialecki , F. J. Brown , J. R. Damewood Jr , C. L. Dantzman , P. D. Edwards , R. T. Jacobs , R. C. Mauger , M. M. Murphy , W. Palmer , K. K. Pine , W. L. Rumsey , L. E. Garcia‐Davenport , A. Shaw , G. B. Steelman , J. M. Surian , E. P. Vacek , Bioorg. Med. Chem. Lett. 2000, 10, 1949. 10987424 10.1016/s0960-894x(00)00387-5

[20] F. J. Moya , J. A. De Juan , A. Ripodas , R. Bernal , A. Fernandez‐Cruz , R. Fernandez‐Durango , Vision. Res. 1998, 38, 3833. 10211376 10.1016/s0042-6989(98)00105-9

[21] W. R. Leifert , G Protein‐Coupled Receptors in Drug Discovery, Humana Press, New York City, NY, 2015, Vol. 552.

[22] S. Locatelli‐Hoops , A. A. Yeliseev , K. Gawrisch , I. Gorshkova , Biomed. Spectrosc. Imaging 2013, 2, 155. 24466506 10.3233/BSI-130045 PMC3898597

[23] C. Yoshiura , Y. Kofuku , T. Ueda , Y. Mase , M. Yokogawa , M. Osawa , Y. Terashima , K. Matsushima , I. Shimada , J. Am. Chem. Soc. 2010, 132, 6768. 20423099 10.1021/ja100830f

[24] N. Bocquet , J. Kohler , M. N. Hug , E. A. Kusznir , A. C. Rufer , R. J. Dawson , M. Hennig , A. Ruf , W. Huber , S. Huber , Biochim. Biophys. Acta ‐ Biomembr. 2015, 1848, 1224. 10.1016/j.bbamem.2015.02.014 25725488

[25] L. Birnbaumer , Cell 1992, 71, 1069. 1335363 10.1016/s0092-8674(05)80056-x

[26] L. Birnbaumer , J. Abramowitz , A. M. Brown , Biochim. Biophys. Acta 1990, 1031, 163. 2160274 10.1016/0304-4157(90)90007-y

[27] M. B. Anand‐Srivastava , P. D. Sehl , D. G. Lowe , J. Biol. Chem. 1996, 271, 19324. 8702617 10.1074/jbc.271.32.19324

[28] F. Fuller , J. G. Porter , A. E. Arfsten , J. Miller , J. W. Schilling , R. M. Scarborough , J. A. Lewicki , D. B. Schenk , J. Biol. Chem. 1988, 263, 9395. 2837487

[29] L. R. Potter , A. R. Yoder , D. R. Flora , L. K. Antos , D. M. Dickey , 2009, 191, 341. 10.1007/978-3-540-68964-5_15 PMC4855512 19089336

[30] F. Mota , C. Fotinou , R. R. Rana , A. W. E. Chan , T. Yelland , M. T. Arooz , A. P. O'Leary , J. Hutton , P. Frankel , I. Zachary , D. Selwood , S. Djordjevic , FEBS J. 2018, 285, 1290. 29430837 10.1111/febs.14405 PMC5947257

[31] J. Powell , F. Mota , D. Steadman , C. Soudy , J. T. Miyauchi , S. Crosby , A. Jarvis , T. Reisinger , N. Winfield , G. Evans , A. Finniear , T. Yelland , Y. T. Chou , A. W. E. Chan , A. O'Leary , L. Cheng , D. Liu , C. Fotinou , C. Milagre , J. F. Martin , H. Jia , P. Frankel , S. Djordjevic , S. E. Tsirka , I. C. Zachary , D. L. Selwood , J. Med. Chem. 2018, 61, 4135. 29648813 10.1021/acs.jmedchem.8b00210 PMC5957473

[32] H. Jia , R. Aqil , L. Cheng , C. Chapman , S. Shaikh , A. Jarvis , A. W. Chan , B. Hartzoulakis , I. M. Evans , A. Frolov , J. Martin , P. Frankel , S. Djordevic , I. C. Zachary , D. L. Selwood , ChemBioChem 2014, 15, 1161. 24771685 10.1002/cbic.201300658

[33] M. Itakura , M. Iwashina , T. Mizuno , T. Ito , H. Hagiwara , S. Hirose , J. Biol. Chem. 1994, 269, 8314. 8132555

[34] X. He , A. Dukkipati , K. C. Garcia , J. Mol. Biol. 2006, 361, 698. 16870210 10.1016/j.jmb.2006.06.060

[35] K. G. Harikumar , E. E. Cawston , L. J. Miller , Assay Drug Dev. Technol. 2011, 9, 394. 21395402 10.1089/adt.2010.0310 PMC3148107

[36] P. Grover , H. Shi , M. Baumgartner , C. J. Camacho , T. E. Smithgall , PLoS ONE 2015, 10, 1. 10.1371/journal.pone.0133590 PMC4519180 26222440

